# Assessment of the Casualty Risk of Multiple Meteorological Hazards in China

**DOI:** 10.3390/ijerph13020222

**Published:** 2016-02-17

**Authors:** Wei Xu, Li Zhuo, Jing Zheng, Yi Ge, Zhihui Gu, Yugang Tian

**Affiliations:** 1 Key Laboratory of Environmental Change and Natural Disasters of Ministry of Education, Beijing Normal University, Beijing 100875, China; xuwei@bnu.edu.cn; 2 Academy of Disaster Reduction and Emergency Management, Ministry of Civil Affairs & Ministry of Education, Beijing Normal University, Beijing 100875, China; 3 Guangdong Provincial Key Laboratory of Urbanization and Geo-simulation, School of Geography and Planning, Sun Yat-Sen University, Guangzhou 510275, China; 4 Guangdong Climate Center, Guangzhou 510080, China; zhengjing@grmc.gov.cn; 5 State Key Laboratory of Pollution Control & Resource Re-use, School of the Environment, Nanjing University, Nanjing 210093, China; geyi@nju.edu.cn; 6 College of Architecture & Urban Planning, Shenzhen University, Shenzhen 518060, China; gzh@szu.edu.cn; 7 School of Information Engineering, China University of Geosciences, Wuhan 430074, China; ygangtian@cug.edu.cn

**Keywords:** meteorological hazard, exceedance probability, risk of multiple hazards, casualty, information diffusion, China

## Abstract

A study of the frequency, intensity, and risk of extreme climatic events or natural hazards is important for assessing the impacts of climate change. Many models have been developed to assess the risk of multiple hazards, however, most of the existing approaches can only model the relative levels of risk. This paper reports the development of a method for the quantitative assessment of the risk of multiple hazards based on information diffusion. This method was used to assess the risks of loss of human lives from 11 types of meteorological hazards in China at the prefectural and provincial levels. Risk curves of multiple hazards were obtained for each province and the risks of 10-year, 20-year, 50-year, and 100-year return periods were mapped. The results show that the provinces (municipalities, autonomous regions) in southeastern China are at higher risk of multiple meteorological hazards as a result of their geographical location and topography. The results of this study can be used as references for the management of meteorological disasters in China. The model can be used to quantitatively calculate the risks of casualty, direct economic losses, building collapse, and agricultural losses for any hazards at different spatial scales.

## 1. Introduction

Evidence of the warming of the Earth’s climate is growing ever stronger [1]. The resulting changes in climate may affect the frequency, intensity, and duration of extreme events, resulting in unprecedented meteorological disasters [2]. An increase in global temperatures may increase the risk of droughts and the intensity of storms, leading to tropical cyclones with higher wind speeds and more intense mid-latitude storms [3]. Since the 1950s, extremely hot days and heavy precipitation have become more common globally and economic losses from climate- and weather-related disasters have increased as a result of large spatial and interannual variations in weather conditions [2,4].

China is one of many countries affected by frequent climate- or weather-related disasters. Examples of such disaster events include the 1998 Yangtze River flood [5] and the extreme storm in Beijing on 21 July 2012 [6]. Losses would be much larger if such extreme events occurred simultaneously at the regional scale and multiple hazards occurred as a result of one another or independently. For example, the 2008 sleet and snow disaster affected 21 provinces (including autonomous regions and municipalities), left 133 people dead or missing, 1.7 million people were displaced and the direct economic losses were more than 151 billion yuan. In this disaster, five sleet and snow events occurred sequentially between 10 January and 6 February [7]. In October 2013, the storm surge caused by Typhoon Fitow combined with high tides inundated the city of Yuyao in Zhejiang Province for several days; more than one million people had to be relocated [8].

Risk assessment is an effective way to reduce the effects of natural hazards and it plays an increasingly important role in disaster management. There are two approaches to the assessment of the risk of multiple hazards. The first approach is to analyze the hazard, exposure, and vulnerability to each hazard separately to obtain indexes for vulnerability and exposure to all hazards. These indexes are then integrated to obtain the risk index of multiple hazards [9,10,11,12]. The second approach is to first calculate the risk for each single hazard independently and then to obtain the risk of multiple hazards by weighing and combining the risks for the single hazards [13,14,15].

These two approaches are frequently used to calculate the risk of multiple hazards. However, the joint probabilities of hazard occurrences and the exceedance probabilities of losses are not considered. This means that these two approaches are often qualitative or semi-quantitative in nature and the results can only be used for a comparative study of regional level risks. Ming *et al.* [16] carried out a quantitative risk assessment of multiple hazards for the loss of crops caused by rainfall and wind to calculate the absolute risk. They determined the vulnerability surface and the joint return period of the hazards in the Yangtze River Delta region. The joint return period of the hazards rainfall and wind was obtained using the Copula method, which can also be used to calculate the joint return period for more than two types of hazards. However, the calculation process is very complicated and the vulnerability curve is difficult to obtain due to limited data on disaster losses. Liu *et al.* [17] carried out a risk assessment of casualty caused jointly by floods and typhoons at the sub-provincial level in the Yangtze River Delta region using an information diffusion method. Xu *et al.* [18] also assessed the risks of building collapse, crop losses, and direct economic losses caused by typhoons and floods at the county level in the same region with the information diffusion method. Based on Xu *et al.* [18], Shi *et al.* [19] assessed the risks jointly caused by floods and typhoons both at the county and the 1 km × 1 km grid scales in the Yangtze River Delta region. Compared with the Copula method, information diffusion has fewer requirements for historical loss data. It should be noted, however, that most of these studies only considered two different hazards. There is a lack of work that quantitatively assesses the risks for more than two hazards.

In the study reported here, the risk of loss of lives—the yearly expected casualties—from 11 different types of meteorological hazards was first assessed and the spatial pattern of the risk was considered at the provincial and the prefectural levels in China using an information diffusion method. The hazards are heavy rain and flood (including waterlogging and storm-induced geological hazards); typhoon (including typhoon-induced storm surges); gale and tornado; thunder and lightning; hail; snow; drought; heat wave; cold spell and freezing; fog; and sand/dust storm.

## 2. Data and Methods

### 2.1. Data and Preprocessing

The data used in this study were the number of historical deaths at the prefectural level caused by the 11 meteorological hazards from 1983 to 2012. These data were obtained from the Ministry of Civil Affairs of China and the China Meteorological Administration.

The interactions of multiple hazards can be classified into four categories: triggering, increasing probability, decreasing probability, and spatiotemporally concurring events [20]. The first three types of interactions have to be carefully explored when assessing the risk of multiple hazards and the resulting models are very complex and require large amounts of specific data. This means that the risk assessment of multiple hazards is difficult to generalize. Risk assessment for events involving spatiotemporally concurring natural hazards is easier because the hazards are independent of each other. Their joint probability can be easily calculated based on the individual probability of each hazard.

Almost all types of natural hazards occur in China and result in great loss of lives every year. In this study, the loss of life from a secondary hazard is grouped under the death toll from the original hazard. For example, the death toll from typhoon-induced floods is grouped under the death toll for typhoons. In this way, the hazards are treated independently; thus the casualty caused by multiple hazards can be simply calculated by summing up the casualty from individual hazards. More than 91,000 people died as a result of meteorological hazards between 1983 and 2012 (Table 1). Heavy rainfall, floods, and the geological hazards induced by these events were the most severe hazards and caused the deaths of 60,932 people (66.79% of the total number of deaths) in this time period. Typhoons and typhoon-induced storm surges caused the deaths of 10,847 people (11.89%). The number of deaths caused by gales and tornados, and thunder and lightning were 6585 and 6344, respectively. The total number of deaths caused by other types of meteorological hazards was 6520 (7.15% of the total death toll).

### 2.2. Methods

**Figure 1:**
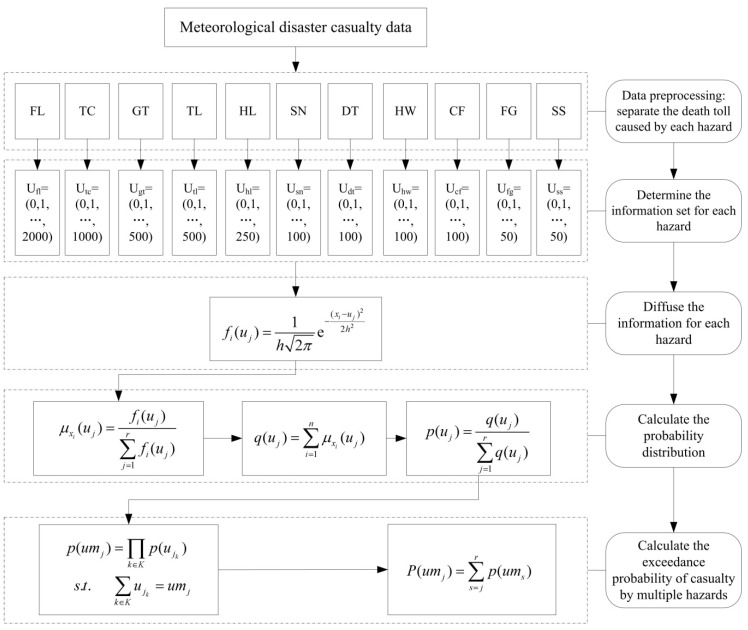
Steps for calculating the exceedance probability of the risk of casualty caused by multiple hazards using an information diffusion method. FL = heavy rain and flood (including waterlogging and storm-induced geological hazards); TC = typhoon (including typhoon-induced storm surges); GT = gales and tornados; TL = thunder and lightning; HL = hail; SN = snow; DT = drought; HW = heat wave; CF = cold spell and freezing; FG = fog; and SS = sand/dust storm. The parameters used in the equations are defined in the text.

Traditional probability models, which require a large data sample, are often unsuitable for quantitative disaster risk assessment due to the limited amount of data on losses. To solve this problem, Huang [21] introduced the information diffusion method to improve the estimated probability. This method has also been applied in the assessment of risk of multiple hazards. For example, Liu *et al.* [17], Xu *et al.* [18], and Shi *et al.* [19] used information diffusion method to assess the risks of casualty, building collapse, crop losses, and direct economic losses jointly caused by floods and typhoons at the sub-provincial level in the Yangtze River Delta region. In this study, we used the information diffusion method to calculate the probability of exceedance of deaths caused by multiple hazards. A flow chart of the method is shown in Figure 1.

Detailed steps for calculating the multiple meteorological hazard risks in China are given in the following sections:

#### *Step 1: define the fuzzy set U* 

Assuming that *x_i_* represents the number of deaths caused by heavy rain and flood (including waterlogging and storm-induced geological hazards) in a province (municipality, autonomous region) for year *i* (*i* = 1,2,…30) and *U_fl_* is the fuzzy set of the number of deaths caused by heavy rain and flood and storm-induced geological hazards in the province. Based on historical flood loss data, *U_fl_* is in the range of 0–2000. The fuzzy set of the number of deaths can then be defined on a discrete universe of discourse:

(1) $$U_{fl}=\{u_{j}|j=1,2,...,r\}=\{u_{1},u_{2},...u_{j}...,u_{2001}\}=\{0,1,2,...,2000\}$$

where *r* is the number of elements in *U_fl_*. In the case of heavy rain and flood, *r* is set as 2001. Similarly, the fuzzy sets for other hazards can be obtained with the number of deaths caused by each hazard falling within a certain range.

#### *Step 2: diffuse information about the number of deaths* 

Information about the number of historical deaths for each year can be diffused to the discrete universe of discourse by applying Equation (2):

(2) $$f_{i}(u_{j})=\frac{1}{h\sqrt{2\pi }}e^{-\frac{{\left(x_{i}-u_{j}\right)}^{2}}{2h^{2}}}$$

where *h* is the diffusion coefficient determined by the minimum value *a*, the maximum value *b*, and the total number *n* of the sample of historical disaster casualties. According to Huang [21], the value of *h* can be calculated by Equation (3) for a given value of *n*:

(3) $$h=\left\{ \begin{array}{l} 0.8146(b-a),\begin{array}{c} 1<n\leq 5, \end{array}\\ 0.5690(b-a),\begin{array}{c} n=6, \end{array}\\ 0.4560(b-a),\begin{array}{c} n=7, \end{array}\\ 0,3860(b-a),\begin{array}{c} n=8, \end{array}\\ 0.3362(b-a),\begin{array}{c} n=9, \end{array}\\ 0.2986(b-a),\begin{array}{c} n=10, \end{array}\\ 2.6851(b-a)/(n-1),\begin{array}{c} n\geq 11. \end{array} \end{array}\right.$$

#### *Step 3: calculate the probability distribution of deaths caused by each hazard* 

The information distribution $\mu_{x_{i}}(u_{j})$ can be obtained after normalizing the value of *f_i_*(*u_j_*), which can be expressed in Equation (4) as a continuous probability density function:

(4) $$\mu_{x_{i}}(u_{j})=\frac{f_{i}(u_{j})}{\sum_{j=1}^{2001}f_{i}(u_{j})}$$

Then the probability distribution *p*(*u_j_*) at *u_j_* (the probability distribution of 0–2000 deaths from heavy rain and flood and storm-induced geological hazards) can be calculated using Equations (5) and (6):

(5) $$q(u_{j})=\sum_{i=1}^{n}\mu_{x_{i}}(u_{j})$$

(6) $$p(u_{j})=\frac{q(u_{j})}{\sum_{j=1}^{2001}q(u_{j})}$$

#### *Step 4: calculate the probability distribution of deaths caused by multiple hazards* 

Assuming that the 11 meteorological hazards occur independently of each other and *K* is the set of these hazards, then the total number of deaths caused by multiple hazards *um_j_* is the sum of the number of deaths caused by the individual hazards. The probability distribution of deaths caused by multiple hazards *p*(*um_j_*) can be calculated by Equations (7) and (8):

(7) $$p(um_{j})=\prod_{k\in K}p(u_{j_{k}})$$

subject to:

(8) $$\sum_{k\in K}u_{j_{k}}=um_{j}$$

where *u_jk_* is the number of deaths caused by hazard *k*.

#### *Step 5: calculate probability of exceedance of deaths caused by multiple hazards* 

The probability of exceedance of human deaths by multiple hazards, *P*(*um_j_*), can be obtained by:

(9) $$P(um_{j})=\sum_{s=j}^{r}p(um_{s})$$

Application of the information diffusion method within the structure of an exceedance probability model gives an improved estimate of the number of deaths due to multiple meteorological hazards and an improved estimation of the absolute risk of multiple meteorological hazards.

## 3. Application and Results

**Figure 2:**
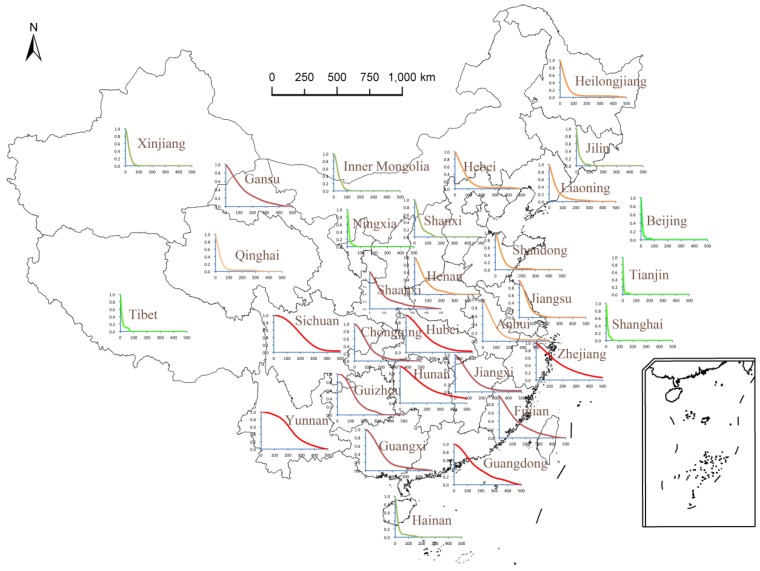
Risk curves of multiple hazards for each province (municipality, autonomous region) in China.

Table 2 and Figure 2 show the results obtained using the multiple hazards model with information diffusion. Figure 2 shows the risk curves for each province (municipality, autonomous region) with the number of deaths on the *x*-axis and the exceedance probability on the *y*-axis. Table 2 gives the exceedance probability for selected death tolls in any year and the average annual death toll, that is, the sum of the area under the risk curve.

The distribution ranges of the number of deaths and the average annual number of deaths vary. Beijing, Tianjin, Shanghai, Ningxia, and Tibet have the narrowest distribution range for the number of deaths (0–100); Shanxi, Hainan, Inner Mongolia, Jilin, and Xinjiang are in the range of 0—200; Anhui, Hubei, and Sichuan are in the range of 0–1000; Zhejiang has a wider range between 0 and 1500; and Gansu has the widest range between 0 and 2000. Other provinces (autonomous regions) are in the range of 0–500.

Figure 2 shows that 13 provinces (municipality, autonomous regions)—including Yunnan, Sichuan, Hunan, Guangdong, Zhejiang, Chongqing, Guizhou, Shaanxi, Hubei, Gansu, Guangxi, Fujian, and Jiangxi—have more than 50% probability of 100 deaths caused by multiple meteorological hazards in any given year. Only Yunnan and Sichuan have >50% probability of 200 deaths caused by multiple hazards in any given year. These provinces (or autonomous regions) are mainly located in southeastern China, which is often affected by floods and typhoons.

The average annual death toll (AADT) of provinces (autonomous regions) in southern China is larger than in the north (Figure 2). Most provinces in the south are at high risk of typhoons, floods, and typhoon- and flood-induced landslides as a result of their geographical location and topography [22,23]. A total of 18 out of 31 provinces (municipalities, autonomous regions) have an AADT <100; Beijing, Tianjin, Shanghai, Ningxia, and Tibet have the lowest AADT of <20, followed by Anhui, Hainan, Jilin, Inner Mongolia, Qinghai, and Xinjiang (20–50). The AADT of Guangdong, Hubei, and Zhejiang is between 150 and 200, while Hunan, Sichuan, and Yunnan are >200.

Yunnan and Sichuan Provinces have the highest risk of rainfall-induced landslides [22,23]. With their dense population, the number of casualties is also fairly large. Therefore these two provinces have the highest AADT. Hunan, Hubei, and Chongqing, with large populations and located in the upper and middle reaches of the Yangtze River, have the highest flood risk. Their AADT values due to multiple meteorological hazards are also very high. Zhejiang, Guangdong, and Fujian are economically developed provinces with large and dense populations along the typhoon-prone eastern and southern coast of China. Therefore, the AADT values are also very high for these provinces.

Although Shanghai and Hainan are located in the typhoon-prone region, their areas and populations are relatively small for a provincial level administrative unit. They have a lower vulnerability to typhoon hazard with regard to human death risk mainly due to their large percentages of urban land and windproof buildings, and well-constructed evacuation shelters [18,19,23]. They also have higher coping capacities to typhoon disasters with well-developed weather forecasting and early warning systems and effective emergency plans [22,23]. Therefore their AADT values are relatively low. Beijing and Tianjin, with low intensity and frequency of hazards, also have low AADT values. The ratio of AADT to regional population was also analyzed. Small population is generally an important contributing factor to lower AADT values, but in Ningxia, Tibet, Xinjiang, and Inner Mongolia where the provincial populations are small [22,23], the AADT to total population ratios are high. Yunnan, Shaanxi, Hunan, Jiangxi, Fujian, Zhejiang, and Chongqing also have high AADT to total population ratios due to their large ADDT values although their total populations are very large too. Sichuan, Guangdong, and Shandong have the lowest AADT to total population ratios because of their large populations.

**Figure 3:**
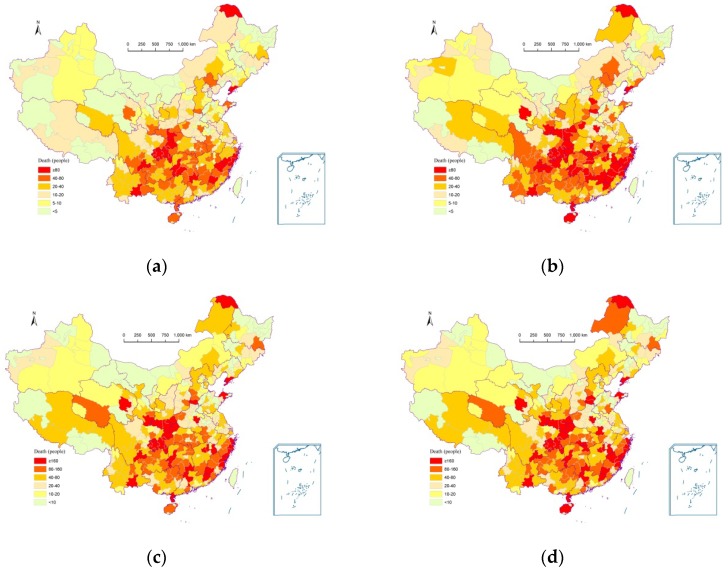
Casualty risks of multiple meteorological hazards with selected exceedance probabilities for each prefecture in China. (**a**) Annual exceedance probability of 10%; (**b**) Annual exceedance probability of 5%; (**c**) Annual exceedance probability of 2%; (**d**) Annual exceedance probability of 1%.

Risk of loss of human lives caused by multiple meteorological hazards for each prefecture in China was also calculated using this method. Figure 3 shows the casualty risks of multiple meteorological hazards for annual exceedance probabilities of 10%, 5%, 2%, and 1%. For any prefecture, the smaller the exceedance probability, the larger the number of deaths.

There was no significant change in the spatial pattern of the number of deaths for these selected exceedance probabilities. Prefectures in southeastern China had higher casualty risks than those in northwestern China as a result of the high intensity of extreme precipitation and typhoons and the high density of population. Areas along the coast and the Yangtze River had particularly high risk of deaths.

**Figure 4:**
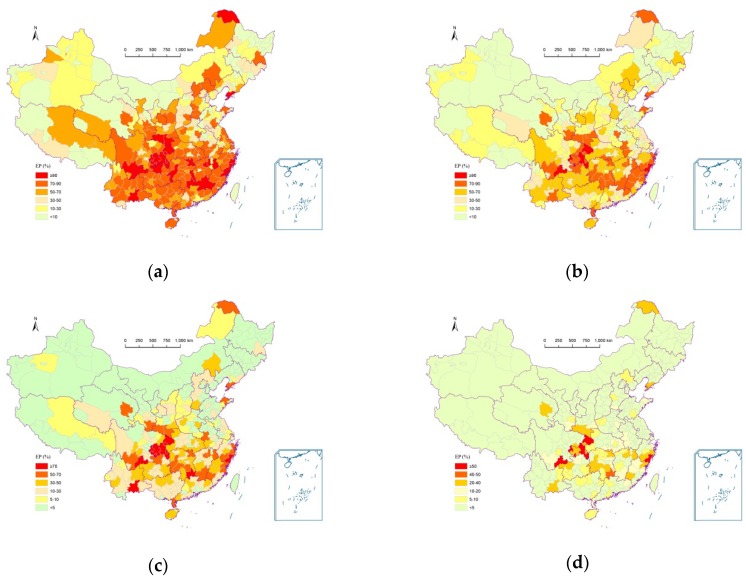
Exceedance probability of selected number of deaths for each prefecture in China. (**a**) Death toll ≥5; (**b**) Death toll ≥10; (**c**) Death toll ≥20; (**d**) Death toll ≥50.

Figure 4 shows the casualty risks of multiple meteorological hazards for selected annual death tolls 5, 10, 20, and 50 for each prefecture in China. The spatial pattern of exceedance probability for a selected number of deaths was similar to that in Figure 3, namely the prefectures in southeastern China had higher exceedance probabilities than those in northwestern China. Areas around the Yangtze River are most at risk in China in term of the risk of casualty caused by multiple meteorological hazards.

## 4. Conclusions

This study quantitatively assessed the risks of 11 meteorological hazards in China at the prefectural and provincial levels using an information diffusion method. The hazards included heavy rain and floods (including waterlogging and storm-induced geological hazards), typhoons (including typhoon-induced storm surges), gales and tornados, thunder and lightning, hail, snow, droughts, heat waves, cold spells and freezing, fog, and sand/dust storms. The results showed that the provinces (municipalities, autonomous regions) in southeastern China have a higher risk of multiple meteorological hazards as a result of the high intensity of extreme precipitation and typhoons due to their geographical location and topography, and the high density of population. A total of 18 out of the 31 provinces (municipalities, autonomous regions) have an AADT value lower than 100. Provinces (municipality, autonomous regions) except Shanghai and Hainan in southeastern China have more than 50% probability of 50 deaths caused by multiple meteorological hazards in any given year. Sichuan and Yunnan with more than 50% probability of 100 deaths have a higher AADT value more than 200. Beijing, Shanghai and Tianjin have lower than 10% probability of 50 deaths in any given year. Prefectures in southeastern China also had higher casualty risks than those in northwestern China as a result of the high intensity of extreme precipitation and typhoons and the high density of population. Areas along the coast and the Yangtze River had a particularly high risk of deaths. 10 prefectures have more than 30% probability of 50 deaths caused by multiple meteorological hazards in any given year. 70 prefectures have the casualty risks of more than 100 deaths by multiple meteorological hazards for annual exceedance probability of 1%.

This is the first study that the risks of loss of lives caused by multiple hazards were quantitatively assessed for different types of meteorological hazards at the prefectural and provincial levels in China using an information diffusion algorithm. This information diffusion model enabled the calculation of the risk of multiple hazards using only limited data. The proposed model can also be used to quantitatively assess risks of loss of lives, direct economic losses, building collapse, and agricultural losses for any type of hazards at different spatial scales. Compared with risk assessment models based on vulnerability curves, the proposed model is very simple and easy to use. In this study, the number of deaths due to secondary hazards was grouped under the primary hazard and therefore it can be assumed that the hazard impacts were independent of each other. If the hazards had been dependent, the Copula method would have been a better choice for the risk analysis of multiple hazards [16]. However, it requires the joint probability of different hazards and the vulnerability curves to be calculated first, which makes the procedure very complicated.

It should also be noted that the multiple hazard risk assessment will be strongly dependent on the quality of the collected data. Although changes in the climate may affect the frequency, intensity, and duration of extreme events, this was not considered in this study. The effects of the regional coping capacity and changes in the population pattern on the risk result were also not considered. For example, with the development of weather forecasting and early warning systems, possible death toll caused by typhoons and typhoon-induced storm surges will be reduced. Growth of population exposure in hazard-prone areas will definitely increase the affected population and/or casualty risks [24]. Integrating scenario analysis, which considers climate change and change of exposure, into the proposed model may be a solution to these problems.

## Figures and Tables

**Table 1:** Loss of human lives caused by meteorological hazards in China, 1983–2012.

Natural Hazard	No. (%) of Deaths
Heavy rain and floods (including waterlogging and storm-induced geological hazards)	60,932 (66.79)
Typhoon (including typhoon-induced storm surge)	10,847 (11.89)
Gale and tornado	6585 (7.22)
Thunder and lightning	6344 (6.95)
Hail	2891 (3.17)
Snow	844 (0.93)
Drought	684 (0.75)
Heat wave	620 (0.68)
Fog	484 (0.53)
Cold spell and freezing	352 (0.39)
Sand/dust storm	131 (0.14)
Other hazards	514 (0.56)

Note: Taiwan, Hong Kong, and Macao are not included due to lack of data.

**Table 2:** Exceedance probability for a given number of deaths for each province (municipality, autonomous region) in China.

Province (Municipality, Autonomous Region)	Exceedance Probability at Different Numbers of Deaths										Average Annual Death Toll
	2	5	10	20	50	100	200	500	1000	1500	
Gansu	0.993	0.983	0.965	0.930	0.820	0.635	0.323	0.042	0.034	0.026	103
Zhejiang	0.994	0.985	0.970	0.938	0.837	0.664	0.384	0.067	0.021	0	155
Hunan	0.996	0.990	0.980	0.957	0.874	0.709	0.427	0.120	0	0	227
Sichuan	0.999	0.998	0.996	0.991	0.963	0.866	0.506	0.035	0	0	220
Jiangxi	0.994	0.984	0.966	0.927	0.786	0.518	0.169	0.030	0	0	130
Hubei	0.996	0.990	0.979	0.954	0.858	0.653	0.290	0.028	0	0	163
Anhui	0.989	0.973	0.944	0.882	0.671	0.344	0.075	0.023	0	0	98
Yunnan	1.000	0.999	0.998	0.996	0.979	0.919	0.633	0	0	0	238
Guangdong	0.998	0.994	0.986	0.968	0.883	0.674	0.357	0	0	0	179
Fujian	0.992	0.980	0.958	0.912	0.760	0.524	0.250	0	0	0	139
Guizhou	0.999	0.998	0.995	0.987	0.924	0.654	0.189	0	0	0	140
Shaanxi	0.990	0.973	0.944	0.881	0.660	0.353	0.172	0	0	0	111
Guangxi	0.996	0.990	0.978	0.949	0.828	0.548	0.159	0	0	0	132
Chongqing	0.996	0.988	0.975	0.940	0.784	0.456	0.127	0	0	0	112
Henan	0.991	0.976	0.948	0.880	0.622	0.300	0.095	0	0	0	86
Hebei	0.987	0.967	0.933	0.857	0.609	0.270	0.046	0	0	0	83
Liaoning	0.975	0.937	0.872	0.737	0.395	0.155	0.045	0	0	0	59
Heilongjiang	0.972	0.930	0.859	0.718	0.361	0.098	0.036	0	0	0	57
Qinghai	0.966	0.913	0.824	0.647	0.247	0.055	0.034	0	0	0	43
Shandong	0.980	0.948	0.891	0.764	0.393	0.099	0.033	0	0	0	53
Jiangsu	0.991	0.975	0.944	0.865	0.556	0.170	0.024	0	0	0	65
Shanxi	0.980	0.947	0.882	0.727	0.304	0.117	0	0	0	0	46
Hainan	0.943	0.854	0.698	0.413	0.082	0.051	0	0	0	0	26
Inner Mongolia	0.991	0.972	0.925	0.767	0.294	0.032	0	0	0	0	40
Jilin	0.920	0.794	0.589	0.313	0.099	0.025	0	0	0	0	21
Xinjiang	0.983	0.948	0.861	0.611	0.140	0.001	0	0	0	0	29
Tibet	0.901	0.736	0.479	0.210	0.103	0	0	0	0	0	16
Ningxia	0.903	0.741	0.481	0.161	0.035	0	0	0	0	0	14
Beijing	0.868	0.666	0.383	0.123	0.034	0	0	0	0	0	12
Tianjin	0.760	0.429	0.119	0.034	0.025	0	0	0	0	0	7
Shanghai	0.908	0.736	0.444	0.157	0.010	0	0	0	0	0	13
